# yrGATE: a web-based gene-structure annotation tool for the identification and dissemination of eukaryotic genes

**DOI:** 10.1186/gb-2006-7-7-r58

**Published:** 2006-07-19

**Authors:** Matthew D Wilkerson, Shannon D Schlueter, Volker Brendel

**Affiliations:** 1 Department of Genetics, Development and Cell Biology, Iowa State University, Ames, IA 50011-3260, USA; 2 Department of Statistics, Iowa State University, Ames, IA 50011-3260, USA

## Abstract

Your Gene structure Annotation Tool for Eukaryotes (yrGATE) provides an Annotation Tool and Community Utilities for worldwide web-based community genome and gene annotation. Annotators can evaluate gene structure evidence derived from multiple sources to create gene structure annotations. Administrators regulate the acceptance of annotations into published gene sets. yrGATE is designed to facilitate rapid and accurate annotation of emerging genomes as well as to confirm, refine, or correct currently published annotations. yrGATE is highly portable and supports different standard input and output formats. The yrGATE software and usage cases are available at .

## Abstract 2

yrGATE is a new web-based tool for community gene and genome annotation.

## Rationale

Complete and accurate gene structure annotation is a prerequisite for the success of many types of genomic projects. For example, gene expression studies based on gene probes would be misleading unless the gene probes uniquely labelled distinct genes. Identification of potential transcription signals relies on correct determination of transcriptional start and termination sites. Characterization of orthologs or paralogs and other studies of molecular phylogeny are also compromised by incomplete or inaccurate gene structure annotation.

Gene structure determination is particularly difficult for eukaryotic genomes. Here, we focus on protein-coding genes. In higher eukaryotes, most of these genes contain introns, and a large fraction of the genes appear to permit alternative splicing [1-3]. High-throughput computational gene structure annotation has been highly successful in providing a first glimpse of the gene content of a genome, but current methods fall short of the goal of complete and accurate gene structure annotation (for example, [4-6]). Recent research has focused on improving prediction sensitivity and specificity by combining multiple sources of evidence [7-9]. However, complexities of transcription and pre-mRNA processing, such as introns in non-coding regions, non-canonical splice sites, and utilization of alternative splice sites, still pose formidable challenges for merely computational methods. Re-annotation efforts for most eukaryotic model genomes have, therefore, relied in large part on manual inspection of gene structure evidence [5,10,11]. However, manual annotation also has shortcomings, such as being typically time-consuming, having exclusive participation, and providing annotations only intermittently [4,10,12].

A policy of 'open annotation', using the internet as the forum for annotation, and bringing annotation into the mainstream has been suggested as a means to eliminate the restraints of manual annotation and to develop high quality gene annotation [13-15]. Several systems have successfully adopted this policy for prokaryote gene annotation (ASAP [16], PeerGAD [17], PseudoCAP [18]). Eukaryotic gene annotation projects have not been able to reap the full benefits of community manual annotation because of the absence of an open online community gene annotation system. Here, we describe newly developed software, Your Gene structure Annotation Tool for Eukaryotes (yrGATE), which seeks to compensate for the inadequacies of traditional manual annotation and to provide a community alternative and/or companion to computational gene annotation, specialized for eukaryotes. yrGATE provides similar functionality as the Apollo annotation tool [19] and NCBI's ModelMaker [20], but includes community utilities, specialized portals to external gene finding and annotation software, and web browser accessibility.

The yrGATE package consists of a web-based Annotation Tool for gene structure annotation creation and Community Utilities for regulating the acceptance of the annotations into a community gene set. The yrGATE Annotation Tool can be used without the Community Utilities for analysis of gene loci independent of a community. The Annotation Tool presents pre-calculated exon evidence in several summaries with different selection mechanisms and provides other methods for specifying custom exons, allowing thorough analysis and quick annotation of loci. Annotators access the tool over the web, where they create an annotation, decide to save the annotation in their personal account, or submit the annotation for review for acceptance into the community gene set. The online nature of yrGATE permits a large and nonexclusive group of annotators, ranging in expertise from professional curators to students [21]. This also provides a continuous timeframe for gene annotation, allowing annotators to examine new sequence evidence as it becomes available and eliminating the delays of periodic annotation. yrGATE is particularly well suited for emerging genomes that are in the process of being sequenced, such as maize. Additionally, the user-friendly character of the yrGATE system contributes to its accessibility and to its potential for community adoption.

## Annotation tool

**Figure 1:**
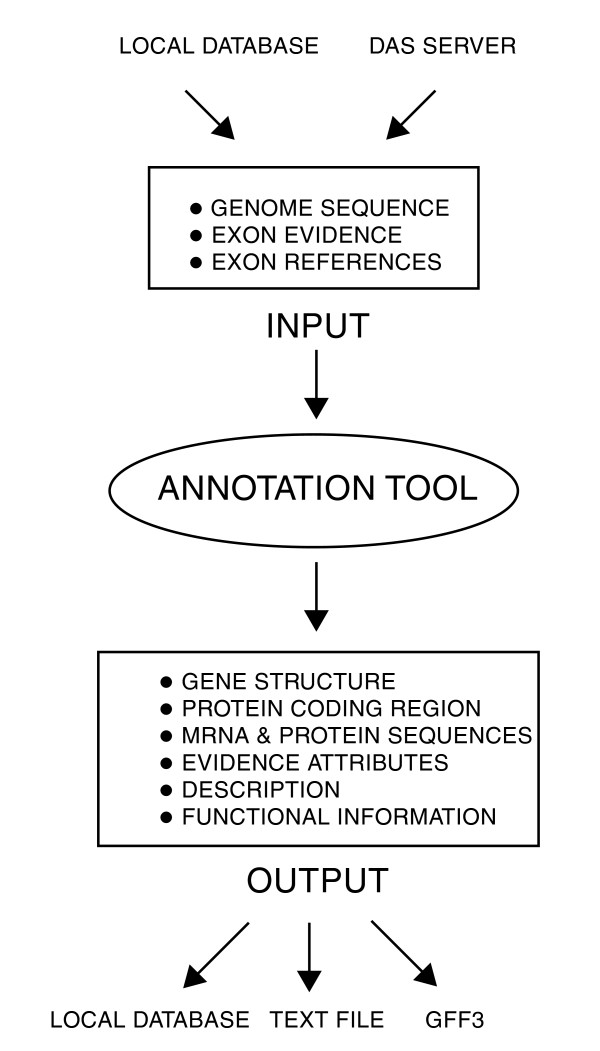
The applications interface of yrGATE. Input to yrGATE is derived from either local database tables or distributed DAS sources. Output is either to local database tables or in the form of simple text or GFF3 files.

The Annotation Tool of the yrGATE package is a web-based utility for creating gene structure annotations. The inputs and outputs of the Annotation Tool are depicted in Figure 1. The input consists of a genomic sequence, exon evidence, and evidence references. The output of the Annotation Tool is a gene annotation, which consists of a gene structure (coordinates of exons and introns), the inferred mRNA sequence, a corresponding protein coding region and its associated translation product, evidence attributes, description, and functional information. The input and output can be in several formats (indicated in Figure 1), which will be described in detail in the Implementation section below.

Defining a gene's exon-intron structure is the central step in creating a eukaryotic gene annotation. The Annotation Tool provides two general categories to specify exons: pre-defined evidence-supported exons and novel user-defined exons. Pre-defined exons are provided by the Annotation Tool from prior computations and are supported by evidence derived from spliced alignments of expressed sequence tags (ESTs) and cDNAs, *ab initio *predictions, or a combination of sources. The evidence is filtered by stringent thresholds to provide exons suggestive of authentic genes. User-defined exons are exons not contained in the pre-defined evidence and are individually specified by the user. Annotators have several channels to designate both categories of exons.

**Figure 2:**
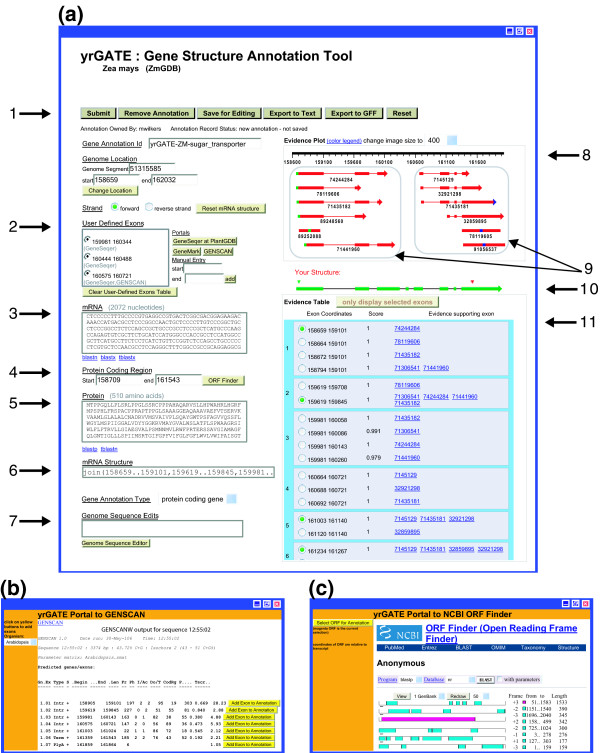
Novel gene annotation. This yrGATE implementation at ZmGDB presents the region 158659-162032 of *Zea mays *BAC gi 51315585. **(a) **The main Annotation Tool window contains a completed gene structure annotation. The provided transcript evidence consists of two groups of ESTs (9, circled) separated by a region with no spanning evidence, 160260-160664 (8). User defined exons have been designated in this region. The User Defined Exons Table (2) lists each exon by coordinates and source. **(b) **Exon 5, 160575..160721, was defined using portals to (b) GENSCAN and GeneSeqer@PlantGDB (not shown). Yellow buttons in the GENSCAN portal (b) add exons to the gene structure in the Annotation Tool (6 in panel a), which are presented pictorially (10 in panel a) for comparison with the Evidence Plot. A protein-coding region was evaluated using the portal to the **(c) **ORF Finder and imported into the Annotation Tool (4 in panel a) using the yellow button.

The Annotation Tool contains three representations of the evidence: the Evidence Plot, the Evidence Table, and links to evidence reference files. The Evidence Plot is a clickable graphic that presents evidence in a color-coded schematic (8 in Figure 2a). The Evidence Table (11 in Figure 2a) groups exons into mutually exclusive groups of exon variants. For each exon, the table lists its genomic coordinates, the maximum score from the method that generated the exon, and the evidence sources that support the exon. The evidence identifiers are hyperlinked to reference files for the exon, which could be an alignment or other program output. Annotators can select pre-defined exons by clicking on exon diagrams in the Evidence Plot or clicking on buttons in the Evidence Table. The annotator's developing gene structure is graphically displayed below the Evidence Plot for visual comparison (10 in Figure 2a).

User-defined exons are specified through portals to exon-generating programs or through entry of the genomic coordinates of an exon. As these exons are defined, they are listed in the User Defined Exons Table (2 in Figure 2a). Acting as a type of web service, portals deliver the genome sequence of the annotation region to an online exon-generating program, with appropriate default parameters specified while allowing the user to change these parameters. The program's output is internally reformatted such that the user can directly add exons from the program's output window into the current gene structure displayed in the yrGATE Annotation Tool window. Currently, portals are available to the gene prediction programs GENSCAN [22] and GeneMark [23] and to the GeneSeqer spliced alignment web server [24]. Administrators can easily add new portals for other exon-generating programs or sequence analysis programs, such as folding programs for non-coding RNA annotations. A template portal is provided with the package.

As an additional channel provided for designating gene structures, the tool allows pasting a coordinate structure into the mRNA structure field (6 in Figure 2a). The format for specifying an mRNA structure follows the conventional notation of designating exons by start and end coordinates separated by non-digits, with multiple exons separated by commas (for example, the Perl regular expression for a two-exon gene structure is [\d+\D+\d+,\d+\D+\d+]). This channel is appropriate for comparing external gene structures with the evidence. Exons not found in the pre-defined evidence are given an 'unknown' source in the User Defined Exons table.

To document the annotator's procedure and parameters, the Exon Origins attribute of an annotation record automatically stores information about the source of each exon. The following information is stored: the method of exon-generation, a score associated with the method and exon, sequence identifiers used in the method, unique database identifiers to the specific output file or record, and a hyperlink to the program output yielding the exon. Exon Origins allows for complete re-creation of the gene structure annotation and for analysis of manual annotation procedures that could aid in future manual annotation efforts and techniques.

After a gene structure has been defined, a user can specify the protein coding region of the annotation through entry of genomic coordinates (4 in Figure 2a) or by using the ORF Finder [20] portal. The ORF Finder portal (Figure 2b), operating similarly to the User Defined Exons portals, allows a user to select an open reading frame, which upon selection is imported into the Annotation Tool window and is graphically represented in the Preview Structure.

Coordinately with gene structure and protein coding region designation and edits, the mRNA and protein sequence fields are updated (3 and 5 in Figure 2a). Hyperlinks, attached to the appropriate sequence, are provided to BLASTN, TBLASTX, BLASTX, TBLASTN and BLASTP at NCBI [20] for an annotator to find similar sequences and/or assign a putative function. Additional pieces of information that can be added to a gene annotation are a description and alternative identifiers.

For cases in which genomic sequence requires editing, such as correction of sequencing errors or annotation of genes undergoing mRNA editing, the Sequence Editor Tool (7 in Figure 2a) enables annotators to insert, delete, or change bases through a web interface. These changes are incorporated into the Annotation Tool and stored with the annotation record.

At the conclusion of a gene annotation session, an annotator decides the outcome of their annotation record (1 in Figure 2a). Annotation records can be saved in the annotator's personal account, which limits access of the annotation to the owner of the annotation. Annotations can be submitted for review, in which case the annotation is sent to administrators, who decide to accept or reject the annotation into a community database for sharing with the community. Alternatively, annotations can be saved locally on the annotator's machine by displaying the annotation in a simple text or GFF3 [25] format. Annotators are also able to delete stored annotations that have not been accepted.

## Community annotation utilities

**Figure 3:**
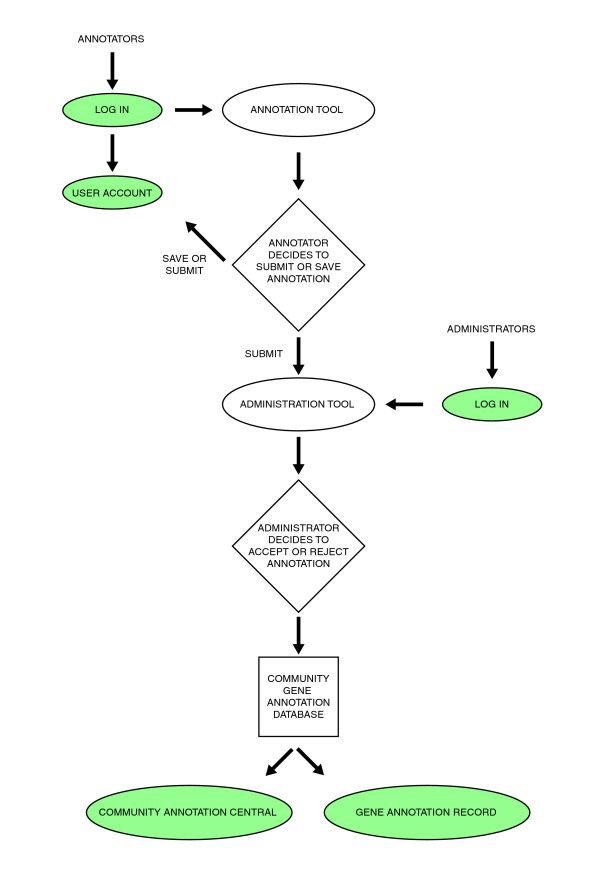
Community annotation review process. Individual Community Utilities are colored green in this diagram.

The yrGATE package includes community annotation utilities for sharing annotations among a public or private community. These utilities form a process for annotation management and review (diagrammed in Figure 3) for two different types of users, annotators and administrators. The types of users are distinguished by their actions: annotators create annotations and administrators review these annotations for acceptance into a community gene set. The community annotation process will be described from the perspective of a new annotation submission and review.

A typical annotation submission begins with an annotator logging in to their private account, which contains all of the annotations created by the annotator. Then, the annotator creates a new annotation using the Annotation Tool and decides to submit the annotation to the community.

This newly submitted annotation is listed in the Administration Tool, where an administrator can 'check out' this annotation for review, so that other administrators do not review this annotation concurrently. The administrator accesses the 'checked-out' annotation in a review version of the Annotation Tool. Then, the administrator reviews the annotation and is able to edit any attributes of the record. When satisfied with their analysis, the administrator accepts or rejects the annotation. If a decision cannot be reached, the annotation is returned to the to-be-reviewed group. Accepted annotations are added to the public community gene annotation database, where they are presented through the Community Annotation Central and Annotation Record facilities. Rejected annotations can be edited by the annotator to be resubmitted for review.

For specific implementations, the described community annotation process can be adjusted by dropping any of the steps, such as eliminating the user log in or eliminating the review process so that all submitted annotations are published. New steps can also be added to the review process, such as a voting utility for submitted annotations.

## Implementations and case studies

The yrGATE package can be implemented in different configurations depending on the input and output (Figure 1) and on the annotation review process (Figure 3). The input can be either from a local database or a DAS server. The output can be an entry in a local database or to a simple text or GFF3 file. The optional Community Utilities provide annotation review and community maintenance facilities. Two yrGATE implementations, having different configurations, are described below.

### Community annotation at PlantGDB

PlantGDB includes a family of species-specific databases: AtGDB [26,27] for *Arabidopsis*, ZmGDB [28] for maize, and OsGDB [29] for rice. These species-specific databases each have an annotation community and an implementation of yrGATE. Input to the yrGATE annotation tool is supplied by the respective PlantGDB database. Pre-calculated exon evidence consists of spliced alignments of EST and cDNA sequences generated by the GeneSeqer program [30]. Evidence references consist of hyperlinks to GeneSeqer output files, which are a part of the respective databases. Genome sequence segments are also supplied by the database. In these PlantGDB implementations, yrGATE Community Utilities regulate user management and annotation curation according to the described default configuration (Figure 3). We illustrate yrGATE usage at PlantGDB with two gene annotation case studies.

The first case study is a novel maize annotation using the ZmGDB yrGATE implementation. An unannotated genome region, 158659-162032 of BAC 51315585, was chosen by the annotator using the genome browsing function of ZmGDB. A screenshot of the Annotation Tool shows the completed annotation (Figure 2). Exons were initially selected from the pre-computed evidence. The evidence, though, consists of two separate groups of ESTs (9 in Figure 2a) with no spanning evidence in the region 160260-160664. The annotator decided to use the GENSCAN and the GeneSeqer@PlantGDB portals to explore potential exons in this region (2 in Figure 2a). After adding three user defined exons, a gene structure connecting both groups of ESTs was defined (6 and 10 in Figure 2a). The portal to the ORF Finder was used to define a protein-coding region, which spanned all eight exons of the putative transcript. Terminal exons, supported by ESTs 71435182 and 32859895, were selected to maximize the untranslated regions. The final step of the annotation session was a BLASTP search at NCBI to compare the novel gene annotation and to assign a putative gene product function. The protein of the annotation had high similarity over most of its length to rice protein NP_915525 and to *Arabidopsis *protein NP_190282. These proteins provided a putative functional assignment of 'sugar transporter' for the annotation. The annotator was satisfied with the annotation and submitted it for review. Administrators reviewed the annotation and accepted it because it was novel and of good quality. The annotation, ZM-yrGATE-sugar_transporter, is now accessible from the ZmGDB Community Annotation Central [31].

**Figure 4:**
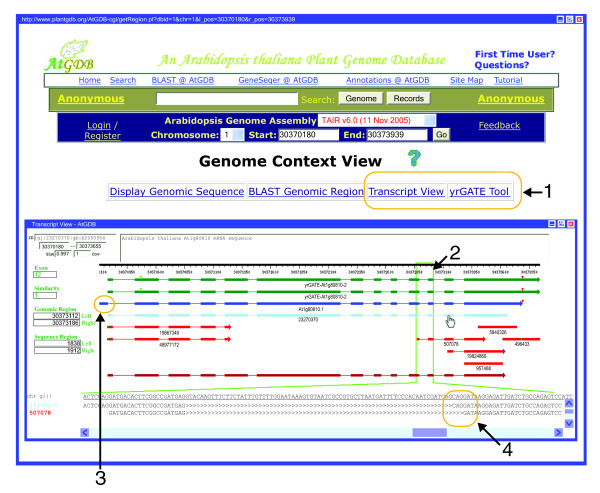
Community implementation of yrGATE at the PlantGDB *Arabidopsis *genome browser, AtGDB, for correction of a public annotation and for alternative splicing. This two-window screenshot depicts yrGATE annotations in the AtGDB browser. The outer window contains a genome context view of AtGDB, which has links to the yrGATE Annotation Tool and to AtGDB's Transcript View (1). The inner window contains the Transcript View, which presents a genome context graphic and sequence alignments represented in the graphic. The graphic has the following color assignments: yrGATE annotations, green; the public annotation, blue; cDNAs, light blue; ESTs, red; annotation protein coding regions, green and red triangles. The multiple sequence alignment in the lower panel of the Transcript View corresponds to the region of graphic contained within the green rectangle (2). The first exon (3) of the public annotation, At1g80810.1, is not supported by expressed sequence evidence, which instead suggests a downstream exon. There are two yrGATE community annotations, yrGATE-At1g80810-1 and yrGATE-At1g80810-2, both of which contain the first exon supported by the evidence but differ at the 3'-end, because the evidence suggests two alternatives for exon 11 (as seen in the multiple alignment display (4)).

The second PlantGDB case study concerns alternative splicing and correction of an inaccurate published annotation of an *Arabidopsis *gene model using the yrGATE implementation at AtGDB. A screenshot of the transcript view of AtGDB presents two accepted community annotations (green structures in interior window, Figure 4). The annotator decided to investigate this genome region (chromosome 1, segment 30370180-30373939) because, upon visual inspection, the first exon of the published annotation At1g808010.1 conflicts with EST and cDNA evidence (3 in Figure 4). Initially, the annotator used cDNA 23270370 to define the gene structure and EST 496433 to extend the 3'-untranslated region. Through the Evidence Table and evidence reference links to GeneSeqer output of the Annotation Tool, the annotator recognized exon 11 has an alternative size supported by EST 507078. The annotator examined open reading frames of both transcript structures, and seeing that both protein-coding regions extend over all exons except for the 5'-most untranslated exon, decided to create two annotations for this locus. An AtGDB administrator reviewed the annotations and accepted both into the community database because they corrected an inaccurate published annotation and captured alternative splicing variants. These alternative splicing variants are displayed in the Transcript View of AtGDB (1 in Figure 4), which displays sequence alignments coordinated to a diagram. In the Transcript View, the green vertical rectangle (2 in Figure 4) relates the diagram to the multiple sequence alignment, where nucleotides in introns are represented by '>' symbols. Comparing alignments for sequences 23270370 and 507078, a three base difference in the start of the exon 11 is apparent (4 in Figure 4). The upstream intron sequences reveal that both intron variants terminate with the standard AG dinucleotide, which suggests this is a probable alternative splicing event. The Transcript View of AtGDB makes such minute differences distinguishable, which were previously concealed in the diagram.

## yrGATE with DAS input

**Figure 5:**
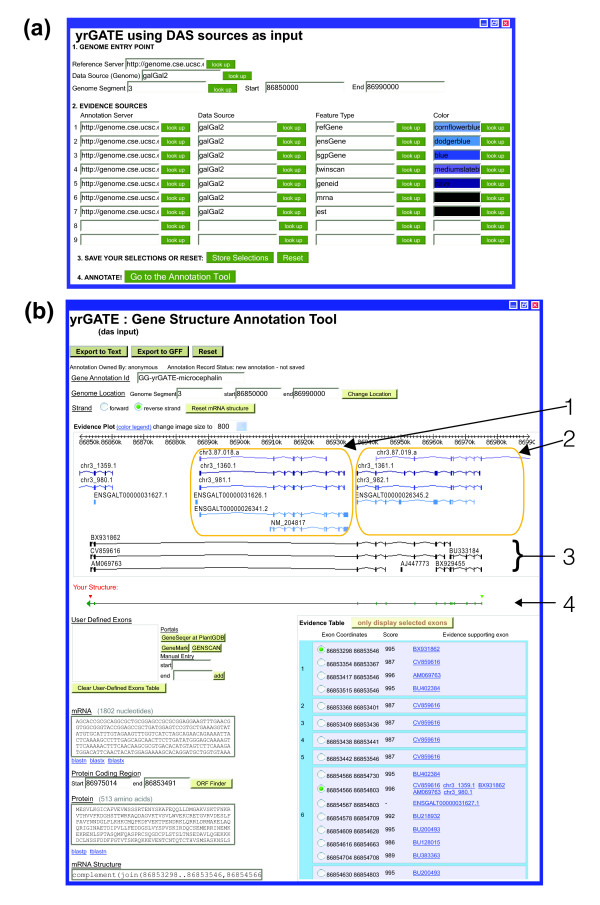
yrGATE with DAS input implementation. **(a) **The entrance to yrGATE is a selection page where a genome and associated evidence sources are specified. Chicken chromosome 3 region 86850000-86990000 is selected. **(b) **EST and mRNA are primary evidence sources (3). Additionally, secondary evidence sources of published annotations are selected for comparison including RefSeq, Ensembl, Twinscan, SGP, and Geneid genes. The novel annotation, GG-yrGATE-microcephalin, is based on EST and mRNA evidence and is distinct from all published chicken annotations in this region on this strand (2). This novel annotation (4) contains a known angiopoietin gene, NM_204817 (1), on the opposite strand within its 12th intron.

DAS servers provide sequence and annotation information that can be queried and is in a standard format [32,33]. The abundance of DAS servers for a variety of organisms provides rich and diverse sources of input for the yrGATE Annotation Tool. An implementation of yrGATE using input data from DAS servers is provided for general use [34]. This implementation, 'yrGATE with DAS input', does not have a community aspect, although a different configuration could add community functionality. The 'yrGATE with DAS input' Selection Page allows an annotator to specify a DAS reference server and DAS evidence sources (Figure 5a). The green 'look up' buttons beside each text box provide a list for annotators to make selections. After these selections are stored, the Annotation Tool can be accessed with the selected input DAS data (Figure 5b).

Figure 5 represents a case study of a novel chicken gene structure annotation. The Selection Page specifies the chicken genome chromosome 3 segment 86850000-86990000 as the genome entry point [35,36]. The selected evidence sources include primary evidence of mRNA and EST BLAT alignments and, for comparison, annotations of types RefSeq [37,38], TWINSCAN [39], Ensembl [40], Geneid [41], and SGP [42]. The published annotation evidence sources are selected so that the annotator can compare primary evidence against existing annotations. Inspection of the primary evidence in the Evidence Plot of the Annotation Tool suggests one gene on the forward strand (approximately 86887000-86934000; 1 in Figure 5b) and another gene on the reverse strand (approximately 86853000-86975000; 2 in Figure 5b). The gene on the forward strand (1 in Figure 5b; for example, RefSeq Gene angiopoietin-2, dark blue, labelled NM_204817.1) is accurately annotated based on mRNA and EST evidence. Additional alternative variants are also accurately annotated.

The primary evidence also suggests an annotation on the reverse strand that contains the angiopoietin-2 gene within one of its introns. However, current annotations on the reverse strand are inaccurate and incomplete based on mRNA and EST evidence (3 in Figure 5b). The first half of this potential gene is represented in some annotations (2 in Figure 5b; SGP, chr3_982.1; Geneid, chr3_1361.1; Ensembl, ENSGALT00000026345.2; TWINSCAN, chr3.87.019.a). Alignments of other species' RefSeq genes [43] (not pictured) indicate a larger gene boundary than the displayed annotations, but this boundary is still too short compared to the primary evidence and does not contain all of the exons supplied by the primary evidence. A novel gene annotation was created on the reverse strand by selecting compatible exons from primary evidence using the Annotation Tool. An open reading frame was designated, and the protein sequence was used to find homologous genes in related species. Based on BLASTP results, this gene was assigned the putative function microcephalin. Interestingly, several species (including human and mouse) have an annotated microcephalin gene with high protein sequence similarity and also maintain the local genome structure of angiopoietin-2 within an intron of the microcephalin gene on the opposite strand.

Links to these case study annotations are provided on the yrGATE website [44].

## Usability and availability

The Annotation Tool was designed with emphasis on usability for annotators. Annotators can immediately select from high quality evidence that has a high likelihood of yielding an accurate annotation and can specify new custom evidence for cases where the evidence is inadequate. The two categories provide for a good annotation process where high quality evidence is first examined and then additional evidence is checked, which is completed in a minimal amount of mouse clicks and screen display, achieved by the tool's design.

The main components of the tool are contained in one standard 1,024 × 768 resolution screen. The tool is loaded once per genomic region, and the form fields are dynamically updated, which allows annotators to quickly evaluate the impact of different exon variants and combinations of exons on the gene structure, mRNA sequence, and protein sequence. yrGATE is compatible with several major operating systems, including Linux, Windows and Macintosh, on several web browsers, of which Mozilla Firefox has the best performance in terms of speed.

yrGATE is available for download [44]. The package consists of Perl, Javascript, HTML, and a MySQL schema. Required Perl libraries for a full implementation are CGI, DBI, LWP, HTTP, PHP::Session, GD, Bio::Graphics, Bio::SeqFeature::Generic, and Bio::Das. Template data are provided for testing and evaluation.

## Conclusion

yrGATE opens gene structure annotation to a large, nonexclusive community. The characteristics of yrGATE contribute to its potential for user appeal and community adoption. Among other applications, it is particularly useful for annotating emerging genomes and for correcting inaccurate published annotations. yrGATE is easily adaptable to different input data and can support a community using the Community Utilities.
